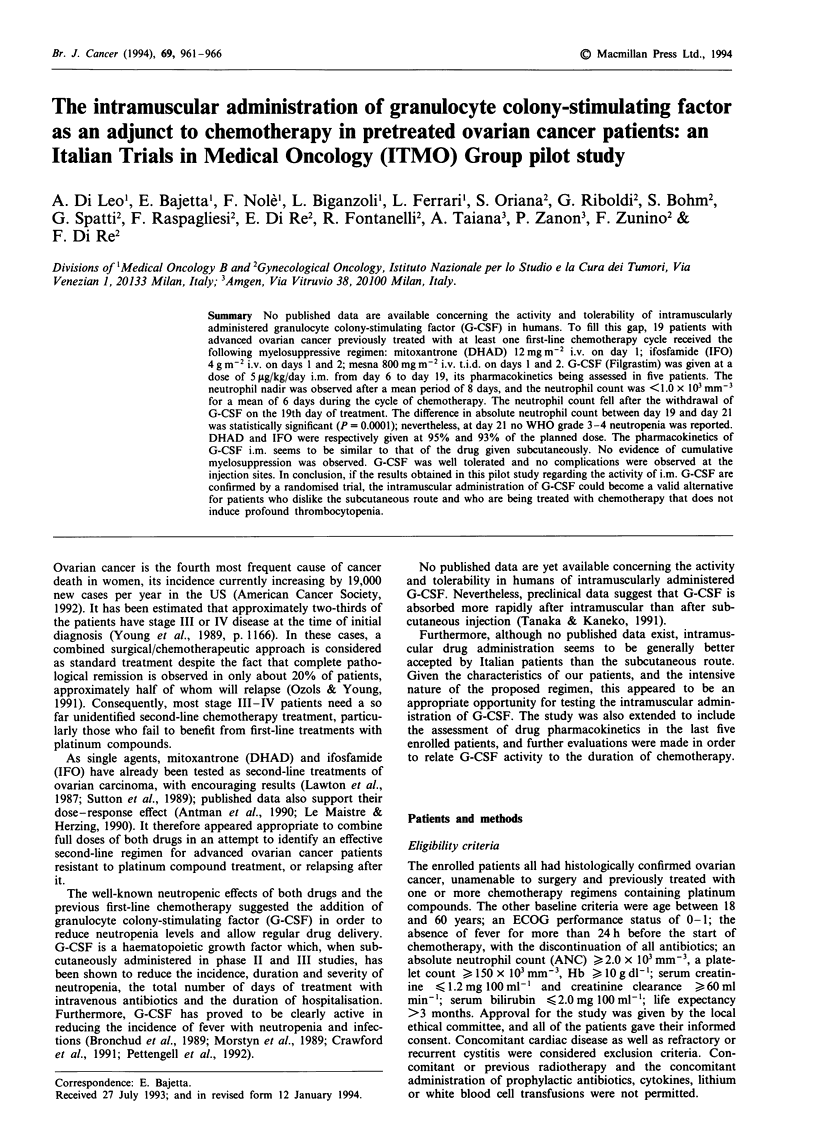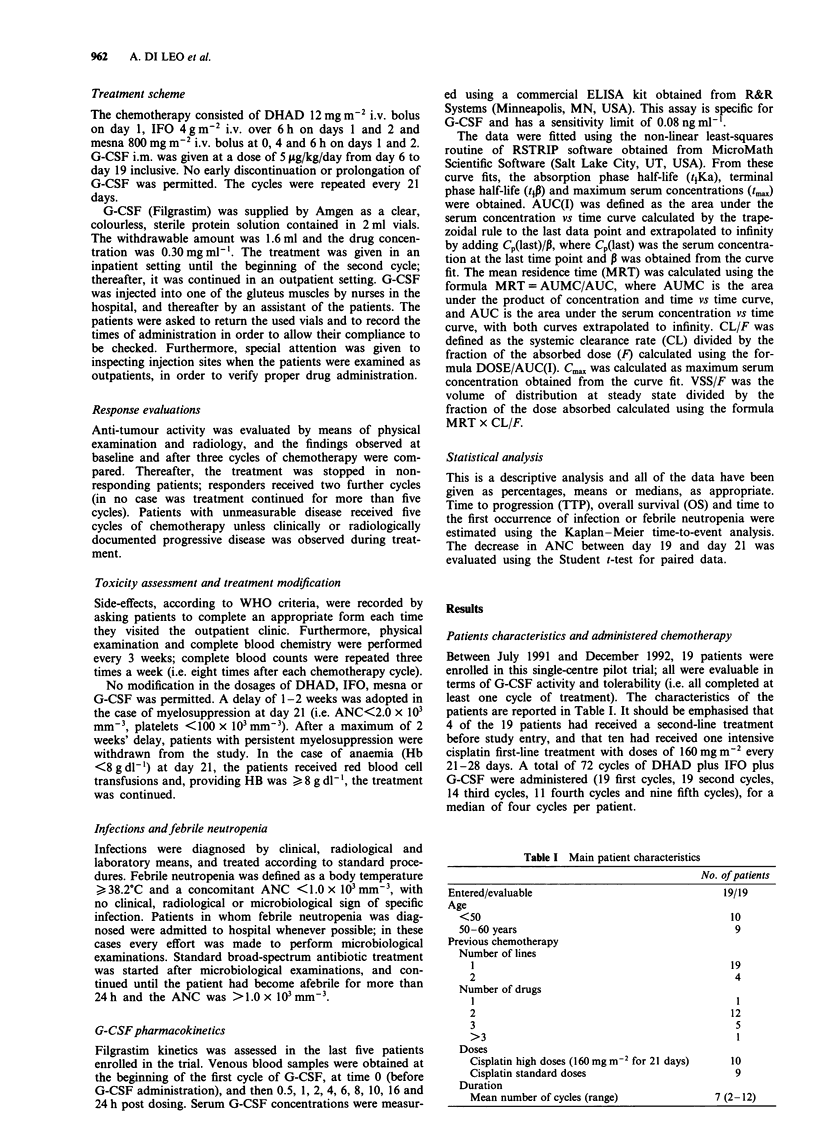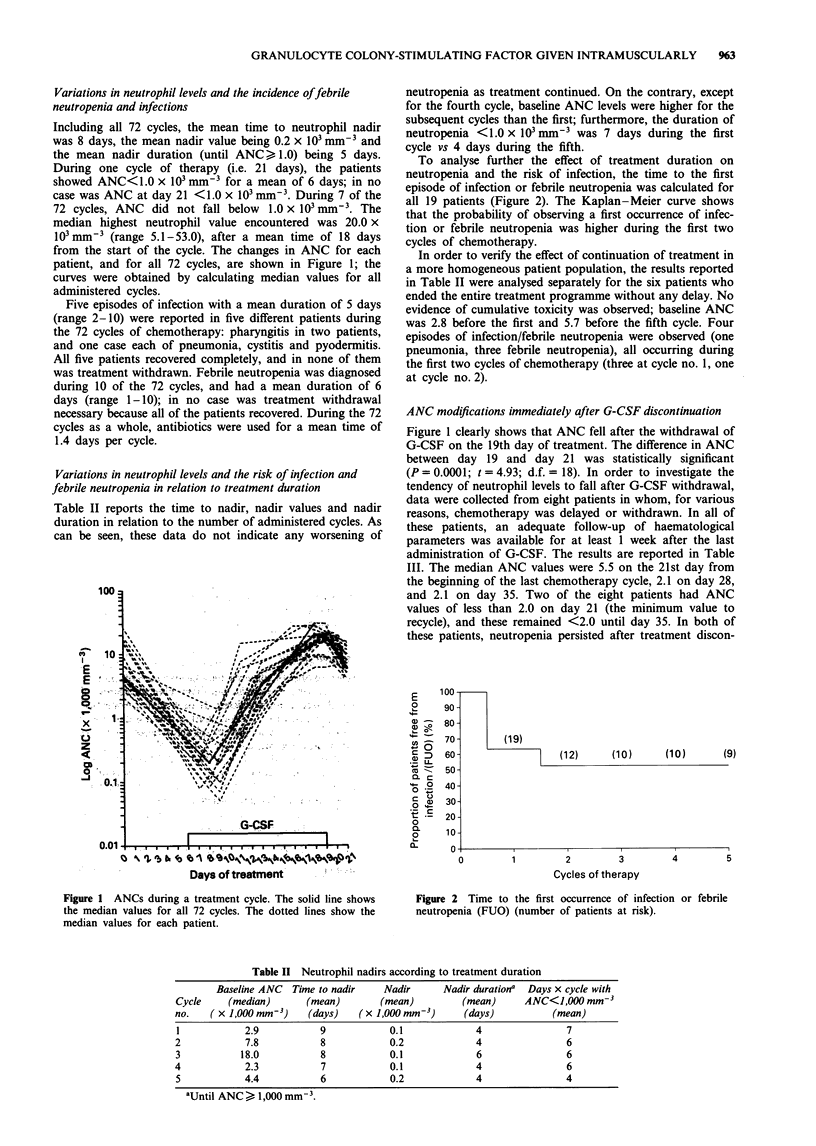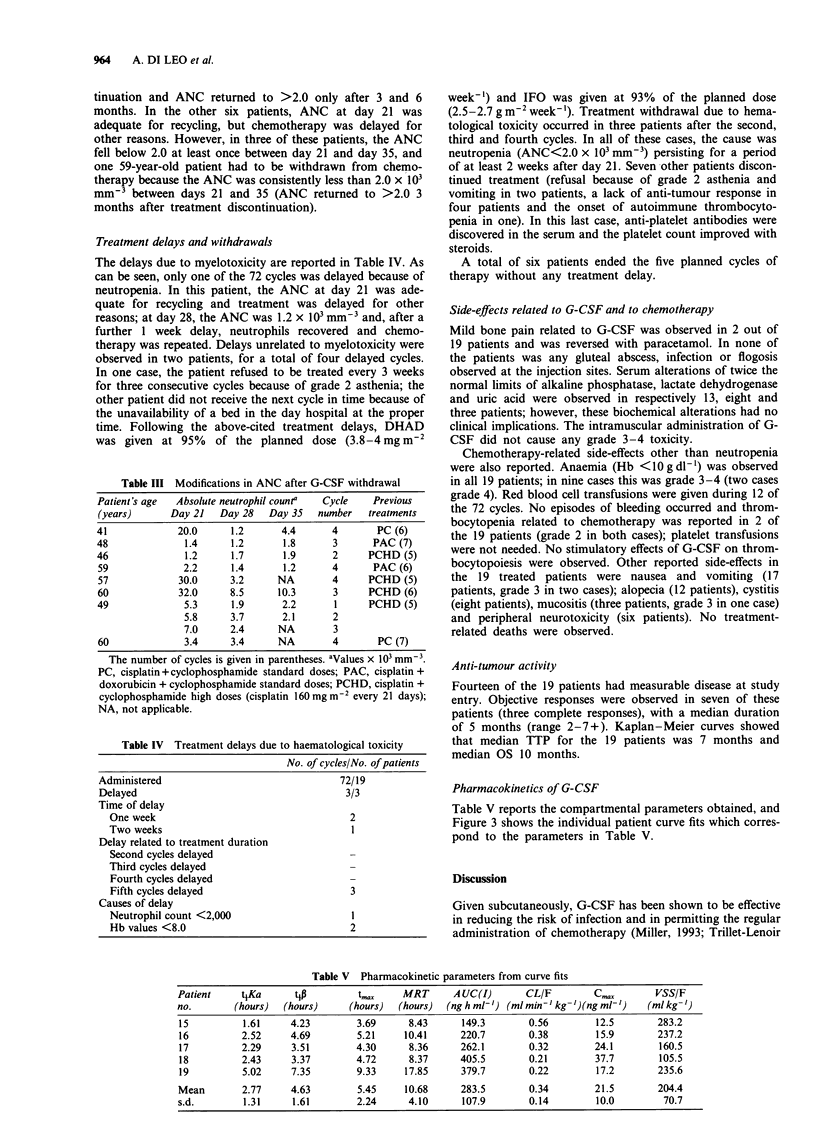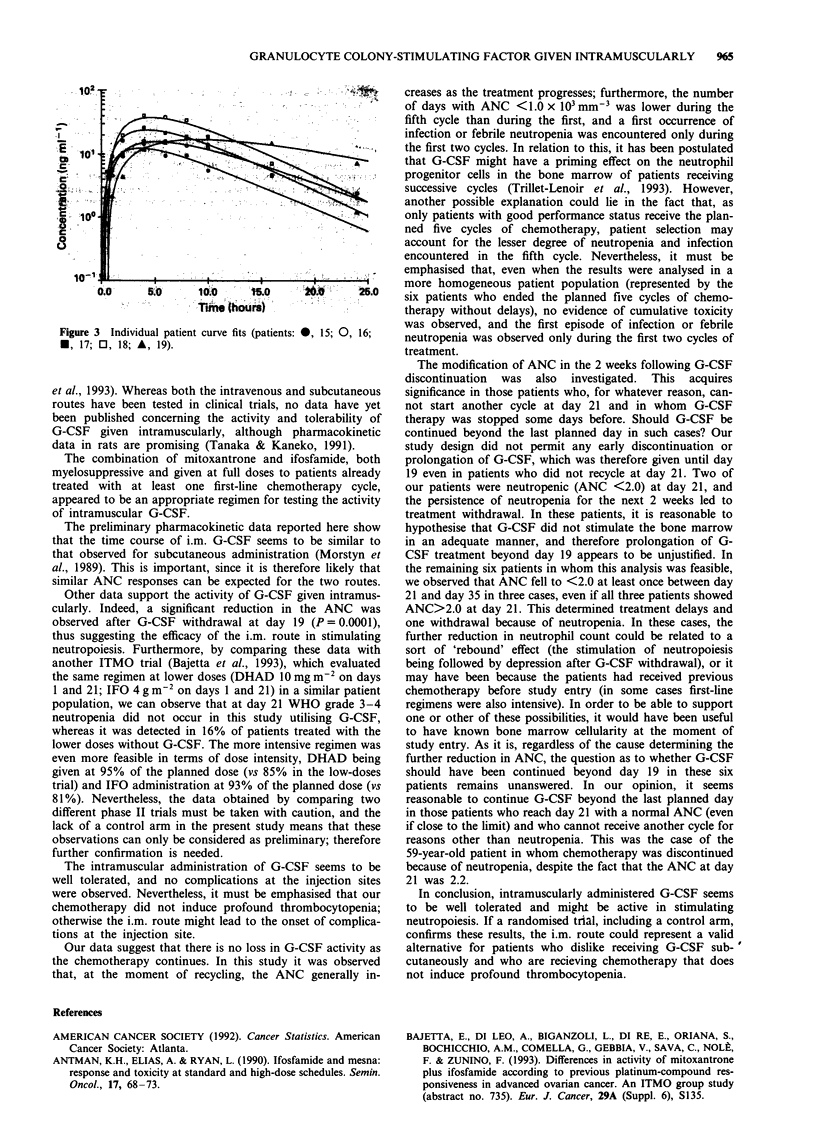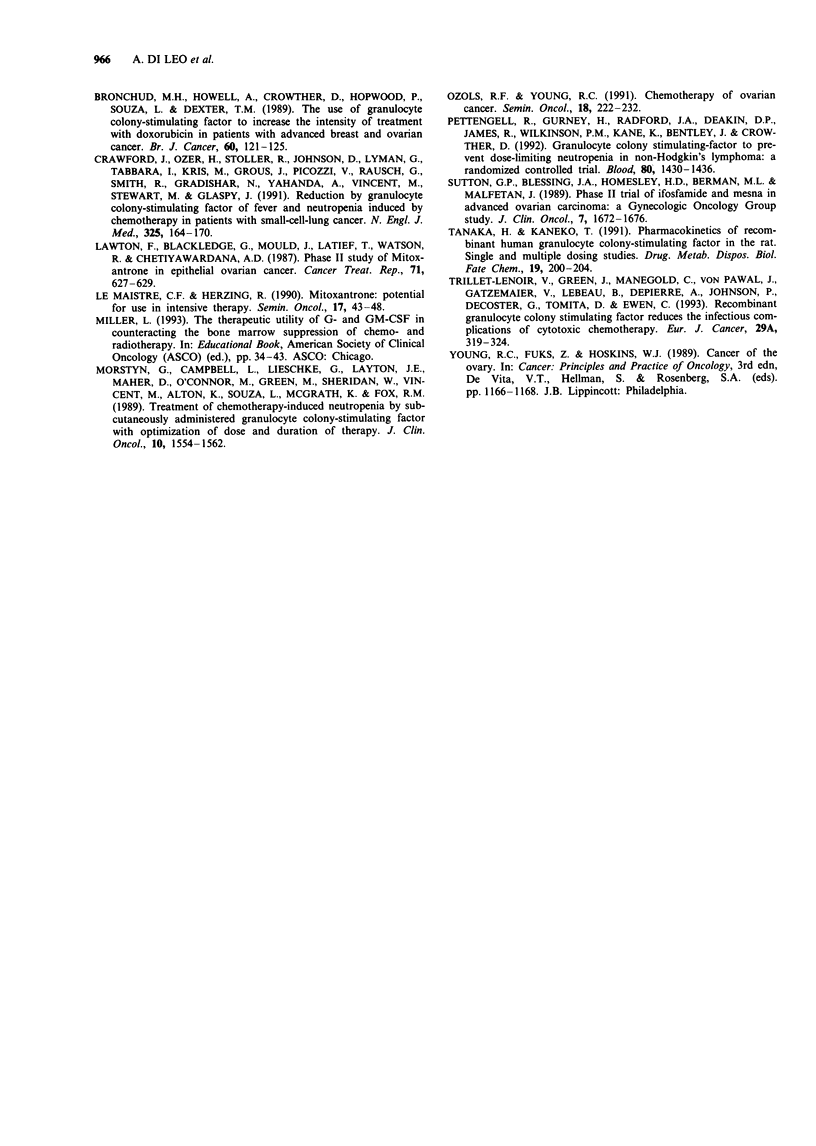# The intramuscular administration of granulocyte colony-stimulating factor as an adjunct to chemotherapy in pretreated ovarian cancer patients: an Italian Trials in Medical Oncology (ITMO) Group pilot study.

**DOI:** 10.1038/bjc.1994.186

**Published:** 1994-05

**Authors:** A. Di Leo, E. Bajetta, F. Nolè, L. Biganzoli, L. Ferrari, S. Oriana, G. Riboldi, S. Bohm, G. Spatti, F. Raspagliesi

**Affiliations:** Division of Medical Oncology B, Istituto Nazionale per lo Studio e la Cura dei Tumori, Milan, Italy.

## Abstract

No published data are available concerning the activity and tolerability of intramuscularly administered granulocyte colony-stimulating factor (G-CSF) in humans. To fill this gap, 19 patients with advanced ovarian cancer previously treated with at least one first-line chemotherapy cycle received the following myelosuppressive regimen: mitoxantrone (DHAD) 12 mg m-2 i.v. on day 1; ifosfamide (IFO) 4 g m-2 i.v. on days 1 and 2; mesna 800 mg m-2 i.v. t.i.d. on days 1 and 2. G-CSF (Filgrastim) was given at a dose of 5 micrograms/kg/day i.m. from day 6 to day 19, its pharmacokinetics being assessed in five patients. The neutrophil nadir was observed after a mean period of 8 days, and the neutrophil count was < 1.0 x 10(3) mm-3 for a mean of 6 days during the cycle of chemotherapy. The neutrophil count fell after the withdrawal of G-CSF on the 19th day of treatment. The difference in absolute neutrophil count between day 19 and day 21 was statistically significant (P = 0.0001); nevertheless, at day 21 no WHO grade 3-4 neutropenia was reported. DHAD and IFO were respectively given at 95% and 93% of the planned dose. The pharmacokinetics of G-CSF i.m. seems to be similar to that of the drug given subcutaneously. No evidence of cumulative myelosuppression was observed. G-CSF was well tolerated and no complications were observed at the injection sites. In conclusion, if the results obtained in this pilot study regarding the activity of i.m. G-CSF are confirmed by a randomised trial, the intramuscular administration of G-CSF could become a valid alternative for patients who dislike the subcutaneous route and who are being treated with chemotherapy that does not induce profound thrombocytopenia.


Br. J. Cancer (1994), 69, 961–966 © Macmillan Press Ltd., 1994

### The intramuscular administration of granulocyte colony-stimulating factor as an adjunct to chemotherapy in pretreated ovarian cancer patients: an Italian Trials in Medical Oncology (ITMO) Group pilot study

A. Di Leo[1], E. Bajetta[1], F. Nolè[1], L. Biganzoli[1], L. Ferrari[1], S. Oriana[2], G. Riboldi[2], S. Bohm[2], G. Spatti[2], F. Raspagliesi[2], E. Di Re[2], R. Fontanelli[2], A. Taiana[3], P. Zanon[3], F. Zunino[2] & F. Di Re[2]

*Divisions of [1]Medical Oncology B and [2]Gynecological Oncology, Istituto Nazionale per lo Studio e la Cura dei Tumori, Via Venezian 1, 20133 Milan, Italy; [3]Amgen, Via Vitruvio 38, 20100 Milan, Italy.*

**Summary** No published data are available concerning the activity and tolerability of intramuscularly administered granulocyte colony-stimulating factor (G-CSF) in humans. To fill this gap, 19 patients with advanced ovarian cancer previously treated with at least one first-line chemotherapy cycle received the following myelosuppressive regimen: mitoxantrone (DHAD) 12 mg $m^{-2}$ i.v. on day 1; ifosfamide (IFO) 4 g $m^{-2}$ i.v. on days 1 and 2; mesna 800 mg $m^{-2}$ i.v. t.i.d. on days 1 and 2. G-CSF (Filgrastim) was given at a dose of 5 μg/kg/day i.m. from day 6 to day 19, its pharmacokinetics being assessed in five patients. The neutrophil nadir was observed after a mean period of 8 days, and the neutrophil count was $<1.0 \times 10^3$ $mm^{-3}$ for a mean of 6 days during the cycle of chemotherapy. The neutrophil count fell after the withdrawal of G-CSF on the 19th day of treatment. The difference in absolute neutrophil count between day 19 and day 21 was statistically significant ($P = 0.0001$); nevertheless, at day 21 no WHO grade 3–4 neutropenia was reported. DHAD and IFO were respectively given at 95% and 93% of the planned dose. The pharmacokinetics of G-CSF i.m. seems to be similar to that of the drug given subcutaneously. No evidence of cumulative myelosuppression was observed. G-CSF was well tolerated and no complications were observed at the injection sites. In conclusion, if the results obtained in this pilot study regarding the activity of i.m. G-CSF are confirmed by a randomised trial, the intramuscular administration of G-CSF could become a valid alternative for patients who dislike the subcutaneous route and who are being treated with chemotherapy that does not induce profound thrombocytopenia.

Ovarian cancer is the fourth most frequent cause of cancer death in women, its incidence currently increasing by 19,000 new cases per year in the US (American Cancer Society, 1992). It has been estimated that approximately two-thirds of the patients have stage III or IV disease at the time of initial diagnosis (Young *et al.*, 1989, p. 1166). In these cases, a combined surgical/chemotherapeutic approach is considered as standard treatment despite the fact that complete pathological remission is observed in only about 20% of patients, approximately half of whom will relapse (Ozols & Young, 1991). Consequently, most stage III–IV patients need a so far unidentified second-line chemotherapy treatment, particularly those who fail to benefit from first-line treatments with platinum compounds.

As single agents, mitoxantrone (DHAD) and ifosfamide (IFO) have already been tested as second-line treatments of ovarian carcinoma, with encouraging results (Lawton *et al.*, 1987; Sutton *et al.*, 1989); published data also support their dose–response effect (Antman *et al.*, 1990; Le Maistre & Herzing, 1990). It therefore appeared appropriate to combine full doses of both drugs in an attempt to identify an effective second-line regimen for advanced ovarian cancer patients resistant to platinum compound treatment, or relapsing after it.

The well-known neutropenic effects of both drugs and the previous first-line chemotherapy suggested the addition of granulocyte colony-stimulating factor (G-CSF) in order to reduce neutropenia levels and allow regular drug delivery. G-CSF is a haematopoietic growth factor which, when subcutaneously administered in phase II and III studies, has been shown to reduce the incidence, duration and severity of neutropenia, the total number of days of treatment with intravenous antibiotics and the duration of hospitalisation. Furthermore, G-CSF has proved to be clearly active in reducing the incidence of fever with neutropenia and infections (Bronchud *et al.*, 1989; Morstyn *et al.*, 1989; Crawford *et al.*, 1991; Pettengell *et al.*, 1992).

Correspondence: E. Bajetta.
Received 27 July 1993; and in revised form 12 January 1994.

No published data are yet available concerning the activity and tolerability in humans of intramuscularly administered G-CSF. Nevertheless, preclinical data suggest that G-CSF is absorbed more rapidly after intramuscular than after subcutaneous injection (Tanaka & Kaneko, 1991).

Furthermore, although no published data exist, intramuscular drug administration seems to be generally better accepted by Italian patients than the subcutaneous route. Given the characteristics of our patients, and the intensive nature of the proposed regimen, this appeared to be an appropriate opportunity for testing the intramuscular administration of G-CSF. The study was also extended to include the assessment of drug pharmacokinetics in the last five enrolled patients, and further evaluations were made in order to relate G-CSF activity to the duration of chemotherapy.

### Patients and methods

### *Eligibility criteria*

The enrolled patients all had histologically confirmed ovarian cancer, unamenable to surgery and previously treated with one or more chemotherapy regimens containing platinum compounds. The other baseline criteria were age between 18 and 60 years; an ECOG performance status of 0–1; the absence of fever for more than 24 h before the start of chemotherapy, with the discontinuation of all antibiotics; an absolute neutrophil count (ANC) $\geqslant 2.0 \times 10^3$ $mm^{-3}$, a platelet count $\geqslant 150 \times 10^3$ $mm^{-3}$, Hb $\geqslant 10$ g $dl^{-1}$; serum creatinine $\leqslant 1.2$ mg 100 $ml^{-1}$ and creatinine clearance $\geqslant 60$ ml $min^{-1}$; serum bilirubin $\leqslant 2.0$ mg 100 $ml^{-1}$; life expectancy >3 months. Approval for the study was given by the local ethical committee, and all of the patients gave their informed consent. Concomitant cardiac disease as well as refractory or recurrent cystitis were considered exclusion criteria. Concomitant or previous radiotherapy and the concomitant administration of prophylactic antibiotics, cytokines, lithium or white blood cell transfusions were not permitted.

*Treatment scheme*

The chemotherapy consisted of DHAD 12 mg $m^{-2}$ i.v. bolus on day 1, IFO 4 g $m^{-2}$ i.v. over 6 h on days 1 and 2 and mesna 800 mg $m^{-2}$ i.v. bolus at 0, 4 and 6 h on days 1 and 2. G-CSF i.m. was given at a dose of 5 μg/kg/day from day 6 to day 19 inclusive. No early discontinuation or prolongation of G-CSF was permitted. The cycles were repeated every 21 days.

G-CSF (Filgrastim) was supplied by Amgen as a clear, colourless, sterile protein solution contained in 2 ml vials. The withdrawable amount was 1.6 ml and the drug concentration was 0.30 mg $ml^{-1}$. The treatment was given in an inpatient setting until the beginning of the second cycle; thereafter, it was continued in an outpatient setting. G-CSF was injected into one of the gluteus muscles by nurses in the hospital, and thereafter by an assistant of the patients. The patients were asked to return the used vials and to record the times of administration in order to allow their compliance to be checked. Furthermore, special attention was given to inspecting injection sites when the patients were examined as outpatients, in order to verify proper drug administration.

*Response evaluations*

Anti-tumour activity was evaluated by means of physical examination and radiology, and the findings observed at baseline and after three cycles of chemotherapy were compared. Thereafter, the treatment was stopped in non-responding patients; responders received two further cycles (in no case was treatment continued for more than five cycles). Patients with unmeasurable disease received five cycles of chemotherapy unless clinically or radiologically documented progressive disease was observed during treatment.

*Toxicity assessment and treatment modification*

Side-effects, according to WHO criteria, were recorded by asking patients to complete an appropriate form each time they visited the outpatient clinic. Furthermore, physical examination and complete blood chemistry were performed every 3 weeks; complete blood counts were repeated three times a week (i.e. eight times after each chemotherapy cycle).

No modification in the dosages of DHAD, IFO, mesna or G-CSF was permitted. A delay of 1–2 weeks was adopted in the case of myelosuppression at day 21 (i.e. ANC $<2.0 \times 10^3$ $mm^{-3}$, platelets $<100 \times 10^3$ $mm^{-3}$). After a maximum of 2 weeks' delay, patients with persistent myelosuppression were withdrawn from the study. In the case of anaemia (Hb $<8$ g $dl^{-1}$) at day 21, the patients received red blood cell transfusions and, providing HB was $\geqslant 8$ g $dl^{-1}$, the treatment was continued.

*Infections and febrile neutropenia*

Infections were diagnosed by clinical, radiological and laboratory means, and treated according to standard procedures. Febrile neutropenia was defined as a body temperature $\geqslant 38.2$°C and a concomitant ANC $<1.0 \times 10^3$ $mm^{-3}$, with no clinical, radiological or microbiological sign of specific infection. Patients in whom febrile neutropenia was diagnosed were admitted to hospital whenever possible; in these cases every effort was made to perform microbiological examinations. Standard broad-spectrum antibiotic treatment was started after microbiological examinations, and continued until the patient had become afebrile for more than 24 h and the ANC was $>1.0 \times 10^3$ $mm^{-3}$.

*G-CSF pharmacokinetics*

Filgrastim kinetics was assessed in the last five patients enrolled in the trial. Venous blood samples were obtained at the beginning of the first cycle of G-CSF, at time 0 (before G-CSF administration), and then 0.5, 1, 2, 4, 6, 8, 10, 16 and 24 h post dosing. Serum G-CSF concentrations were measured using a commercial ELISA kit obtained from R&R Systems (Minneapolis, MN, USA). This assay is specific for G-CSF and has a sensitivity limit of 0.08 ng $ml^{-1}$.

The data were fitted using the non-linear least-squares routine of RSTRIP software obtained from MicroMath Scientific Software (Salt Lake City, UT, USA). From these curve fits, the absorption phase half-life ($t_{\frac{1}{2}}$Ka), terminal phase half-life ($t_{\frac{1}{2}}\beta$) and maximum serum concentrations ($t_{max}$) were obtained. AUC(I) was defined as the area under the serum concentration *vs* time curve calculated by the trapezoidal rule to the last data point and extrapolated to infinity by adding $C_p$(last)/$\beta$, where $C_p$(last) was the serum concentration at the last time point and $\beta$ was obtained from the curve fit. The mean residence time (MRT) was calculated using the formula MRT = AUMC/AUC, where AUMC is the area under the product of concentration and time *vs* time curve, and AUC is the area under the serum concentration *vs* time curve, with both curves extrapolated to infinity. CL/*F* was defined as the systemic clearance rate (CL) divided by the fraction of the absorbed dose (*F*) calculated using the formula DOSE/AUC(I). $C_{max}$ was calculated as maximum serum concentration obtained from the curve fit. VSS/*F* was the volume of distribution at steady state divided by the fraction of the dose absorbed calculated using the formula MRT × CL/*F*.

*Statistical analysis*

This is a descriptive analysis and all of the data have been given as percentages, means or medians, as appropriate. Time to progression (TTP), overall survival (OS) and time to the first occurrence of infection or febrile neutropenia were estimated using the Kaplan–Meier time-to-event analysis. The decrease in ANC between day 19 and day 21 was evaluated using the Student *t*-test for paired data.

### Results

*Patients characteristics and administered chemotherapy*

Between July 1991 and December 1992, 19 patients were enrolled in this single-centre pilot trial; all were evaluable in terms of G-CSF activity and tolerability (i.e. all completed at least one cycle of treatment). The characteristics of the patients are reported in Table I. It should be emphasised that 4 of the 19 patients had received a second-line treatment before study entry, and that ten had received one intensive cisplatin first-line treatment with doses of 160 mg $m^{-2}$ every 21–28 days. A total of 72 cycles of DHAD plus IFO plus G-CSF were administered (19 first cycles, 19 second cycles, 14 third cycles, 11 fourth cycles and nine fifth cycles), for a median of four cycles per patient.

**Table I** Main patient characteristics

| | *No. of patients* |
|---|---|
| Entered/evaluable | 19/19 |
| Age | |
| $<50$ | 10 |
| 50–60 years | 9 |
| Previous chemotherapy | |
| Number of lines | |
| 1 | 19 |
| 2 | 4 |
| Number of drugs | |
| 1 | 1 |
| 2 | 12 |
| 3 | 5 |
| $>3$ | 1 |
| Doses | |
| Cisplatin high doses (160 mg $m^{-2}$ for 21 days) | 10 |
| Cisplatin standard doses | 9 |
| Duration | |
| Mean number of cycles (range) | 7 (2–12) |

*Variations in neutrophil levels and the incidence of febrile neutropenia and infections*

Including all 72 cycles, the mean time to neutrophil nadir was 8 days, the mean nadir value being $0.2 \times 10^3\,mm^{-3}$ and the mean nadir duration (until ANC$\geqslant$1.0) being 5 days. During one cycle of therapy (i.e. 21 days), the patients showed ANC$<1.0 \times 10^3\,mm^{-3}$ for a mean of 6 days; in no case was ANC at day 21 $<1.0 \times 10^3\,mm^{-3}$. During 7 of the 72 cycles, ANC did not fall below $1.0 \times 10^3\,mm^{-3}$. The median highest neutrophil value encountered was $20.0 \times 10^3\,mm^{-3}$ (range 5.1–53.0), after a mean time of 18 days from the start of the cycle. The changes in ANC for each patient, and for all 72 cycles, are shown in Figure 1; the curves were obtained by calculating median values for all administered cycles.

Five episodes of infection with a mean duration of 5 days (range 2–10) were reported in five different patients during the 72 cycles of chemotherapy: pharyngitis in two patients, and one case each of pneumonia, cystitis and pyodermitis. All five patients recovered completely, and in none of them was treatment withdrawn. Febrile neutropenia was diagnosed during 10 of the 72 cycles, and had a mean duration of 6 days (range 1–10); in no case was treatment withdrawal necessary because all of the patients recovered. During the 72 cycles as a whole, antibiotics were used for a mean time of 1.4 days per cycle.

*Variations in neutrophil levels and the risk of infection and febrile neutropenia in relation to treatment duration*

Table II reports the time to nadir, nadir values and nadir duration in relation to the number of administered cycles. As can be seen, these data do not indicate any worsening of neutropenia as treatment continued. On the contrary, except for the fourth cycle, baseline ANC levels were higher for the subsequent cycles than the first; furthermore, the duration of neutropenia $<1.0 \times 10^3\,mm^{-3}$ was 7 days during the first cycle *vs* 4 days during the fifth.

To analyse further the effect of treatment duration on neutropenia and the risk of infection, the time to the first episode of infection or febrile neutropenia was calculated for all 19 patients (Figure 2). The Kaplan–Meier curve shows that the probability of observing a first occurrence of infection or febrile neutropenia was higher during the first two cycles of chemotherapy.

In order to verify the effect of continuation of treatment in a more homogeneous patient population, the results reported in Table II were analysed separately for the six patients who ended the entire treatment programme without any delay. No evidence of cumulative toxicity was observed; baseline ANC was 2.8 before the first and 5.7 before the fifth cycle. Four episodes of infection/febrile neutropenia were observed (one pneumonia, three febrile neutropenia), all occurring during the first two cycles of chemotherapy (three at cycle no. 1, one at cycle no. 2).

*ANC modifications immediately after G-CSF discontinuation*

Figure 1 clearly shows that ANC fell after the withdrawal of G-CSF on the 19th day of treatment. The difference in ANC between day 19 and day 21 was statistically significant ($P = 0.0001$; $t = 4.93$; d.f. = 18). In order to investigate the tendency of neutrophil levels to fall after G-CSF withdrawal, data were collected from eight patients in whom, for various reasons, chemotherapy was delayed or withdrawn. In all of these patients, an adequate follow-up of haematological parameters was available for at least 1 week after the last administration of G-CSF. The results are reported in Table III. The median ANC values were 5.5 on the 21st day from the beginning of the last chemotherapy cycle, 2.1 on day 28, and 2.1 on day 35. Two of the eight patients had ANC values of less than 2.0 on day 21 (the minimum value to recycle), and these remained <2.0 until day 35. In both of these patients, neutropenia persisted after treatment discon-

**Figure 1** ANCs during a treatment cycle. The solid line shows the median values for all 72 cycles. The dotted lines show the median values for each patient.

**Figure 2** Time to the first occurrence of infection or febrile neutropenia (FUO) (number of patients at risk).

**Table II** Neutrophil nadirs according to treatment duration

| Cycle no. | *Baseline ANC (median) (× 1,000 mm⁻³)* | *Time to nadir (mean) (days)* | *Nadir (mean) (× 1,000 mm⁻³)* | *Nadir duration[a] (mean) (days)* | *Days × cycle with ANC<1,000 mm⁻³ (mean)* |
|---|---|---|---|---|---|
| 1 | 2.9 | 9 | 0.1 | 4 | 7 |
| 2 | 7.8 | 8 | 0.2 | 4 | 6 |
| 3 | 18.0 | 8 | 0.1 | 6 | 6 |
| 4 | 2.3 | 7 | 0.1 | 4 | 6 |
| 5 | 4.4 | 6 | 0.2 | 4 | 4 |

[a]Until ANC$\geqslant$1,000 $mm^{-3}$.

tinuation and ANC returned to >2.0 only after 3 and 6 months. In the other six patients, ANC at day 21 was adequate for recycling, but chemotherapy was delayed for other reasons. However, in three of these patients, the ANC fell below 2.0 at least once between day 21 and day 35, and one 59-year-old patient had to be withdrawn from chemotherapy because the ANC was consistently less than $2.0 \times 10^3$ $mm^{-3}$ between days 21 and 35 (ANC returned to >2.0 3 months after treatment discontinuation).

*Treatment delays and withdrawals*

The delays due to myelotoxicity are reported in Table IV. As can be seen, only one of the 72 cycles was delayed because of neutropenia. In this patient, the ANC at day 21 was adequate for recycling and treatment was delayed for other reasons; at day 28, the ANC was $1.2 \times 10^3\ mm^{-3}$ and, after a further 1 week delay, neutrophils recovered and chemotherapy was repeated. Delays unrelated to myelotoxicity were observed in two patients, for a total of four delayed cycles. In one case, the patient refused to be treated every 3 weeks for three consecutive cycles because of grade 2 asthenia; the other patient did not receive the next cycle in time because of the unavailability of a bed in the day hospital at the proper time. Following the above-cited treatment delays, DHAD was given at 95% of the planned dose ($3.8-4\ mg\ m^{-2}$ $week^{-1}$) and IFO was given at 93% of the planned dose ($2.5-2.7\ g\ m^{-2}\ week^{-1}$). Treatment withdrawal due to hematological toxicity occurred in three patients after the second, third and fourth cycles. In all of these cases, the cause was neutropenia ($ANC<2.0 \times 10^3\ mm^{-3}$) persisting for a period of at least 2 weeks after day 21. Seven other patients discontinued treatment (refusal because of grade 2 asthenia and vomiting in two patients, a lack of anti-tumour response in four patients and the onset of autoimmune thrombocytopenia in one). In this last case, anti-platelet antibodies were discovered in the serum and the platelet count improved with steroids.

A total of six patients ended the five planned cycles of therapy without any treatment delay.

**Table III** Modifications in ANC after G-CSF withdrawal

| *Patient's age (years)* | *Absolute neutrophil count*[a] *Day 21* | *Day 28* | *Day 35* | *Cycle number* | *Previous treatments* |
|---|---|---|---|---|---|
| 41 | 20.0 | 1.2 | 4.4 | 4 | PC (6) |
| 48 | 1.4 | 1.2 | 1.8 | 3 | PAC (7) |
| 46 | 1.2 | 1.7 | 1.9 | 2 | PCHD (5) |
| 59 | 2.2 | 1.4 | 1.2 | 4 | PAC (6) |
| 57 | 30.0 | 3.2 | NA | 4 | PCHD (5) |
| 60 | 32.0 | 8.5 | 10.3 | 3 | PCHD (6) |
| 49 | 5.3 | 1.9 | 2.2 | 1 | PCHD (5) |
| | 5.8 | 3.7 | 2.1 | 2 | |
| | 7.0 | 2.4 | NA | 3 | |
| 60 | 3.4 | 3.4 | NA | 4 | PC (7) |

The number of cycles is given in parentheses. [a]Values $\times 10^3\ mm^{-3}$. PC, cisplatin + cyclophosphamide standard doses; PAC, cisplatin + doxorubicin + cyclophosphamide standard doses; PCHD, cisplatin + cyclophosphamide high doses (cisplatin $160\ mg\ m^{-2}$ every 21 days); NA, not applicable.

**Table IV** Treatment delays due to haematological toxicity

| | *No. of cycles/No. of patients* |
|---|---|
| Administered | 72/19 |
| Delayed | 3/3 |
| Time of delay | |
| One week | 2 |
| Two weeks | 1 |
| Delay related to treatment duration | |
| Second cycles delayed | – |
| Third cycles delayed | – |
| Fourth cycles delayed | – |
| Fifth cycles delayed | 3 |
| Causes of delay | |
| Neutrophil count <2,000 | 1 |
| Hb values <8.0 | 2 |

*Side-effects related to G-CSF and to chemotherapy*

Mild bone pain related to G-CSF was observed in 2 out of 19 patients and was reversed with paracetamol. In none of the patients was any gluteal abscess, infection or flogosis observed at the injection sites. Serum alterations of twice the normal limits of alkaline phosphatase, lactate dehydrogenase and uric acid were observed in respectively 13, eight and three patients; however, these biochemical alterations had no clinical implications. The intramuscular administration of G-CSF did not cause any grade 3–4 toxicity.

Chemotherapy-related side-effects other than neutropenia were also reported. Anaemia (Hb $<10\ g\ dl^{-1}$) was observed in all 19 patients; in nine cases this was grade 3–4 (two cases grade 4). Red blood cell transfusions were given during 12 of the 72 cycles. No episodes of bleeding occurred and thrombocytopenia related to chemotherapy was reported in 2 of the 19 patients (grade 2 in both cases); platelet transfusions were not needed. No stimulatory effects of G-CSF on thrombocytopoiesis were observed. Other reported side-effects in the 19 treated patients were nausea and vomiting (17 patients, grade 3 in two cases); alopecia (12 patients), cystitis (eight patients), mucositis (three patients, grade 3 in one case) and peripheral neurotoxicity (six patients). No treatment-related deaths were observed.

*Anti-tumour activity*

Fourteen of the 19 patients had measurable disease at study entry. Objective responses were observed in seven of these patients (three complete responses), with a median duration of 5 months (range 2–7+). Kaplan–Meier curves showed that median TTP for the 19 patients was 7 months and median OS 10 months.

*Pharmacokinetics of G-CSF*

Table V reports the compartmental parameters obtained, and Figure 3 shows the individual patient curve fits which correspond to the parameters in Table V.

### Discussion

Given subcutaneously, G-CSF has been shown to be effective in reducing the risk of infection and in permitting the regular administration of chemotherapy (Miller, 1993; Trillet-Lenoir

**Table V** Pharmacokinetic parameters from curve fits

| *Patient no.* | $t_{\frac{1}{2}}Ka$ *(hours)* | $t_{\frac{1}{2}}\beta$ *(hours)* | $t_{max}$ *(hours)* | *MRT (hours)* | *AUC(I)* ($ng\ h\ ml^{-1}$) | *CL/F* ($ml\ min^{-1}\ kg^{-1}$) | $C_{max}$ ($ng\ ml^{-1}$) | *VSS/F* ($ml\ kg^{-1}$) |
|---|---|---|---|---|---|---|---|---|
| 15 | 1.61 | 4.23 | 3.69 | 8.43 | 149.3 | 0.56 | 12.5 | 283.2 |
| 16 | 2.52 | 4.69 | 5.21 | 10.41 | 220.7 | 0.38 | 15.9 | 237.2 |
| 17 | 2.29 | 3.51 | 4.30 | 8.36 | 262.1 | 0.32 | 24.1 | 160.5 |
| 18 | 2.43 | 3.37 | 4.72 | 8.37 | 405.5 | 0.21 | 37.7 | 105.5 |
| 19 | 5.02 | 7.35 | 9.33 | 17.85 | 379.7 | 0.22 | 17.2 | 235.6 |
| Mean | 2.77 | 4.63 | 5.45 | 10.68 | 283.5 | 0.34 | 21.5 | 204.4 |
| s.d. | 1.31 | 1.61 | 2.24 | 4.10 | 107.9 | 0.14 | 10.0 | 70.7 |

**Figure 3** Individual patient curve fits (patients: ●, 15; ○, 16; ■, 17; □, 18; ▲, 19).

*et al.*, 1993). Whereas both the intravenous and subcutaneous routes have been tested in clinical trials, no data have yet been published concerning the activity and tolerability of G-CSF given intramuscularly, although pharmacokinetic data in rats are promising (Tanaka & Kaneko, 1991).

The combination of mitoxantrone and ifosfamide, both myelosuppressive and given at full doses to patients already treated with at least one first-line chemotherapy cycle, appeared to be an appropriate regimen for testing the activity of intramuscular G-CSF.

The preliminary pharmacokinetic data reported here show that the time course of i.m. G-CSF seems to be similar to that observed for subcutaneous administration (Morstyn *et al.*, 1989). This is important, since it is therefore likely that similar ANC responses can be expected for the two routes.

Other data support the activity of G-CSF given intramuscularly. Indeed, a significant reduction in the ANC was observed after G-CSF withdrawal at day 19 ($P = 0.0001$), thus suggesting the efficacy of the i.m. route in stimulating neutropoiesis. Furthermore, by comparing these data with another ITMO trial (Bajetta *et al.*, 1993), which evaluated the same regimen at lower doses (DHAD 10 mg $m^{-2}$ on days 1 and 21; IFO 4 g $m^{-2}$ on days 1 and 21) in a similar patient population, we can observe that at day 21 WHO grade 3–4 neutropenia did not occur in this study utilising G-CSF, whereas it was detected in 16% of patients treated with the lower doses without G-CSF. The more intensive regimen was even more feasible in terms of dose intensity, DHAD being given at 95% of the planned dose (*vs* 85% in the low-doses trial) and IFO administration at 93% of the planned dose (*vs* 81%). Nevertheless, the data obtained by comparing two different phase II trials must be taken with caution, and the lack of a control arm in the present study means that these observations can only be considered as preliminary; therefore further confirmation is needed.

The intramuscular administration of G-CSF seems to be well tolerated, and no complications at the injection sites were observed. Nevertheless, it must be emphasised that our chemotherapy did not induce profound thrombocytopenia; otherwise the i.m. route might lead to the onset of complications at the injection site.

Our data suggest that there is no loss in G-CSF activity as the chemotherapy continues. In this study it was observed that, at the moment of recycling, the ANC generally increases as the treatment progresses; furthermore, the number of days with ANC $<1.0 \times 10^3$ $mm^{-3}$ was lower during the fifth cycle than during the first, and a first occurrence of infection or febrile neutropenia was encountered only during the first two cycles. In relation to this, it has been postulated that G-CSF might have a priming effect on the neutrophil progenitor cells in the bone marrow of patients receiving successive cycles (Trillet-Lenoir *et al.*, 1993). However, another possible explanation could lie in the fact that, as only patients with good performance status receive the planned five cycles of chemotherapy, patient selection may account for the lesser degree of neutropenia and infection encountered in the fifth cycle. Nevertheless, it must be emphasised that, even when the results were analysed in a more homogeneous patient population (represented by the six patients who ended the planned five cycles of chemotherapy without delays), no evidence of cumulative toxicity was observed, and the first episode of infection or febrile neutropenia was observed only during the first two cycles of treatment.

The modification of ANC in the 2 weeks following G-CSF discontinuation was also investigated. This acquires significance in those patients who, for whatever reason, cannot start another cycle at day 21 and in whom G-CSF therapy was stopped some days before. Should G-CSF be continued beyond the last planned day in such cases? Our study design did not permit any early discontinuation or prolongation of G-CSF, which was therefore given until day 19 even in patients who did not recycle at day 21. Two of our patients were neutropenic (ANC $<2.0$) at day 21, and the persistence of neutropenia for the next 2 weeks led to treatment withdrawal. In these patients, it is reasonable to hypothesise that G-CSF did not stimulate the bone marrow in an adequate manner, and therefore prolongation of G-CSF treatment beyond day 19 appears to be unjustified. In the remaining six patients in whom this analysis was feasible, we observed that ANC fell to $<2.0$ at least once between day 21 and day 35 in three cases, even if all three patients showed ANC$>$2.0 at day 21. This determined treatment delays and one withdrawal because of neutropenia. In these cases, the further reduction in neutrophil count could be related to a sort of 'rebound' effect (the stimulation of neutropoiesis being followed by depression after G-CSF withdrawal), or it may have been because the patients had received previous chemotherapy before study entry (in some cases first-line regimens were also intensive). In order to be able to support one or other of these possibilities, it would have been useful to have known bone marrow cellularity at the moment of study entry. As it is, regardless of the cause determining the further reduction in ANC, the question as to whether G-CSF should have been continued beyond day 19 in these six patients remains unanswered. In our opinion, it seems reasonable to continue G-CSF beyond the last planned day in those patients who reach day 21 with a normal ANC (even if close to the limit) and who cannot receive another cycle for reasons other than neutropenia. This was the case of the 59-year-old patient in whom chemotherapy was discontinued because of neutropenia, despite the fact that the ANC at day 21 was 2.2.

In conclusion, intramuscularly administered G-CSF seems to be well tolerated and might be active in stimulating neutropoiesis. If a randomised trial, including a control arm, confirms these results, the i.m. route could represent a valid alternative for patients who dislike receiving G-CSF subcutaneously and who are recieving chemotherapy that does not induce profound thrombocytopenia.

### References

AMERICAN CANCER SOCIETY (1992). *Cancer Statistics*. American Cancer Society: Atlanta.

ANTMAN, K.H., ELIAS, A. & RYAN, L. (1990). Ifosfamide and mesna: response and toxicity at standard and high-dose schedules. *Semin. Oncol.*, **17**, 68–73.

BAJETTA, E., DI LEO, A., BIGANZOLI, L., DI RE, E., ORIANA, S., BOCHICCHIO, A.M., COMELLA, G., GEBBIA, V., SAVA, C., NOLÈ, F. & ZUNINO, F. (1993). Differences in activity of mitoxantrone plus ifosfamide according to previous platinum-compound responsiveness in advanced ovarian cancer. An ITMO group study (abstract no. 735). *Eur. J. Cancer*, **29A** (Suppl. 6), S135.

BRONCHUD, M.H., HOWELL, A., CROWTHER, D., HOPWOOD, P., SOUZA, L. & DEXTER, T.M. (1989). The use of granulocyte colony-stimulating factor to increase the intensity of treatment with doxorubicin in patients with advanced breast and ovarian cancer. *Br. J. Cancer*, **60**, 121-125.

CRAWFORD, J., OZER, H., STOLLER, R., JOHNSON, D., LYMAN, G., TABBARA, I., KRIS, M., GROUS, J., PICOZZI, V., RAUSCH, G., SMITH, R., GRADISHAR, N., YAHANDA, A., VINCENT, M., STEWART, M. & GLASPY, J. (1991). Reduction by granulocyte colony-stimulating factor of fever and neutropenia induced by chemotherapy in patients with small-cell-lung cancer. *N. Engl. J. Med.*, **325**, 164-170.

LAWTON, F., BLACKLEDGE, G., MOULD, J., LATIEF, T., WATSON, R. & CHETIYAWARDANA, A.D. (1987). Phase II study of Mitoxantrone in epithelial ovarian cancer. *Cancer Treat. Rep.*, **71**, 627-629.

LE MAISTRE, C.F. & HERZING, R. (1990). Mitoxantrone: potential for use in intensive therapy. *Semin. Oncol.*, **17**, 43-48.

MILLER, L. (1993). The therapeutic utility of G- and GM-CSF in counteracting the bone marrow suppression of chemo- and radiotherapy. In: *Educational Book*, American Society of Clinical Oncology (ASCO) (ed.), pp. 34-43. ASCO: Chicago.

MORSTYN, G., CAMPBELL, L., LIESCHKE, G., LAYTON, J.E., MAHER, D., O'CONNOR, M., GREEN, M., SHERIDAN, W., VINCENT, M., ALTON, K., SOUZA, L., MCGRATH, K. & FOX, R.M. (1989). Treatment of chemotherapy-induced neutropenia by subcutaneously administered granulocyte colony-stimulating factor with optimization of dose and duration of therapy. *J. Clin. Oncol.*, **10**, 1554-1562.

OZOLS, R.F. & YOUNG, R.C. (1991). Chemotherapy of ovarian cancer. *Semin. Oncol.*, **18**, 222-232.

PETTENGELL, R., GURNEY, H., RADFORD, J.A., DEAKIN, D.P., JAMES, R., WILKINSON, P.M., KANE, K., BENTLEY, J. & CROWTHER, D. (1992). Granulocyte colony stimulating-factor to prevent dose-limiting neutropenia in non-Hodgkin's lymphoma: a randomized controlled trial. *Blood*, **80**, 1430-1436.

SUTTON, G.P., BLESSING, J.A., HOMESLEY, H.D., BERMAN, M.L. & MALFETAN, J. (1989). Phase II trial of ifosfamide and mesna in advanced ovarian carcinoma: a Gynecologic Oncology Group study. *J. Clin. Oncol.*, **7**, 1672-1676.

TANAKA, H. & KANEKO, T. (1991). Pharmacokinetics of recombinant human granulocyte colony-stimulating factor in the rat. Single and multiple dosing studies. *Drug. Metab. Dispos. Biol. Fate Chem.*, **19**, 200-204.

TRILLET-LENOIR, V., GREEN, J., MANEGOLD, C., VON PAWAL, J., GATZEMAIER, V., LEBEAU, B., DEPIERRE, A., JOHNSON, P., DECOSTER, G., TOMITA, D. & EWEN, C. (1993). Recombinant granulocyte colony stimulating factor reduces the infectious complications of cytotoxic chemotherapy. *Eur. J. Cancer*, **29A**, 319-324.

YOUNG, R.C., FUKS, Z. & HOSKINS, W.J. (1989). Cancer of the ovary. In: *Cancer: Principles and Practice of Oncology*, 3rd edn, De Vita, V.T., Hellman, S. & Rosenberg, S.A. (eds). pp. 1166-1168. J.B. Lippincott: Philadelphia.